# Predator cues and environmental complexity shape the behaviour and life history of juvenile lobsters (*Homarus gammarus*)

**DOI:** 10.1098/rsos.241940

**Published:** 2025-05-14

**Authors:** Giovanni Polverino, Lorenzo Latini, Giuseppe Nascetti, Giacomo Grignani, Eleonora Bello, Claudia Gili, Claudio Carere, Daniele Canestrelli

**Affiliations:** ^1^ Ecological and Biological Sciences, University of Tuscia, Viterbo, Italy; ^2^ School of Biological Sciences, Monash University, Clayton, Victoria, Australia; ^3^ Stazione Zoologica Anton Dohrn, Napoli, Italy

**Keywords:** behavioural plasticity, conservation aquaculture, developmental plasticity, environmental enrichment, risk taking, crustaceans

## Abstract

Phenotypic plasticity is critical for animals to survive in our rapidly changing world, especially for species with low population densities. Standard hatchery procedures can assist some conservation programmes by producing large numbers of juveniles to be released into the wild. Yet we know surprisingly little about the impact that such standard, minimalistic hatchery settings have on the development of important behaviours and life-history traits of the animals. Here, we fill this gap by testing whether and how early-life exposure to different environmental conditions alters the development of ecologically relevant behaviours and life-history traits of the European lobster (*Homarus gammarus*)—one of the most harvested species in the Mediterranean. We used the progeny of wild-caught females and manipulated—in a full factorial design—the environmental complexity of the individual enclosures and the level of perceived predation risk. We repeatedly quantified key behaviours (activity, shelter use and aggressiveness) and life-history traits (carapace length and intermoult period) of individuals throughout their early development, capturing both mean and individual-level variation across treatments. Our results offer solid evidence that standard hatchery settings compromise the development of important behavioural and life-history traits of lobsters and even alter their behavioural plasticity—probably reducing the effectiveness of conservation programmes.

## 1. Introduction

In conservation aquaculture, aquatic organisms are typically reproduced in captivity in large numbers for their reintroduction into the wild, with the aim of restoring depleted populations and enhancing wild stocks [1]. However, conventional hatchery-rearing procedures impose different selection pressures on the individuals relative to their natural environment, potentially altering the development of those phenotypic traits that are critical for these organisms to survive in the wild [2–4].

Marine decapod crustaceans are commonly included into stock enhancement programmes, because these organisms have high fecundity but relatively low recruitment rates, i.e. high larval mortality in the wild, making such programmes particularly valuable for restoring populations that are heavily harvested [5] and/or that suffer from other anthropogenic pressures (e.g. chemical pollution). Marine decapods also play a fundamental role in marine trophic webs, but most of their natural populations, especially prawns (e.g. *Penaeus japonicus*, *Penaeus chinensis*), crabs (e.g. *Scylla paramamosain*, *Portunus trituberculatus*), and lobsters (e.g. *Homarus gammarus*), have been overfished in the past and are currently managed for their conservation to guarantee a sustainable exploitation [6–8]. So the release of farmed crustaceans has risen over the past few decades, due to the increase in their demand for food consumption and, therefore, their market value [9]. Breeding these organisms requires capturing ovigerous females in the wild and maintaining them in controlled, safe environments until the eggs hatch so that the offspring are released into the wild only after they have passed the most vulnerable stages of their life, i.e. the planktonic larval and the soft-shelled juvenile stage [6]. However, classic rearing conditions are unable to guarantee a wild-like development of the individuals because they are designed to optimize growth and reproduction rather than mimic the challenging conditions from the wild [4]. From a stock enhancement perspective, producing animals that are not capable of facing changing environments is likely to result in poor conservation programmes. Indeed, the post-release ecological competence of the animals reared in aquaculture settings is not obvious [4]. Improper rearing conditions could impair feeding and interspecific (e.g. predator avoidance, parasite infections, competition) and intraspecific interactions (e.g. dominance, courtship, mating) and even compromise immune functions, sexual and defence competences, cognitive abilities and claws differentiation [4,10,11].

Evidence suggests that early-life conditions experienced by an individual play a key role in shaping the development of its phenotype in adulthood, including its capacity to adjust its behaviour to face environmental challenges [12–14]. A large portion of behavioural diversity and plasticity within animal groups can be, in fact, attributed to the effect of different environmental conditions experienced by the individuals early in their lives [15,16]. However, the role of environmental effects during ontogeny as proximate mechanisms for shaping individual differences in behaviour is often overlooked—but see some of the first empirical works on this topic [17–19]. It is therefore important to understand whether and how environmental heterogeneity can contribute, at least in the short term, to individual differences in behavioural plasticity. For example, in addition to improving animal welfare [20,21], ecologically relevant cues introduced into hatchery-rearing environments could also benefit conservation programmes by promoting diverse coping strategies in animals and a greater investment in their phenotypic plasticity [5,22–24]. Shedding light on these aspects is critical for managing risks and ensuring the effectiveness of conservation programmes [2,25].

Here, we aimed to test whether and how the early-life exposure to different environmental conditions alters the development of ecologically relevant behaviours and life-history traits of hatchery-reared juvenile European lobsters (*H. gammarus*), an ecologically and economically important decapod species, historically targeted for conservation programmes. Specifically, 256 juvenile lobsters were exposed to different degrees of environmental complexity during their early benthic stages, which included the presence/absence of a shelter and substrate into their individual rearing enclosures and the exposure to cues from their natural predators. Juveniles were repeatedly assayed one by one during their early development for ecologically relevant behaviours (activity, shelter use and aggressiveness [26]) and life-history traits (carapace length and intermoult period [27]). This approach allowed us to test for treatment effects on both mean and individual-level variation in key behaviours and life-history traits [28]. In accordance with previous studies on *H. gammarus* (e.g. [29–34]), we hypothesized that standard, minimalistic aquaculture settings would compromise the development of complex behavioural repertoires of the animals and alter their growth (see also [4] and references therein). Furthermore, we expected that effects of standard hatchery conditions on lobsters’ development would extend beyond mean changes in their behaviour and life history, impairing also the capacity of the animals to adjust their phenotypes to rapid changes of their surrounding environment (e.g. predation risk [35]). Our analysis sheds light on the role of early-life environmental stimuli in shaping mean and individual-level variation in animal phenotypes and also offers important insights for science-based improvements in aquaculture practices and conservation programmes.

## 2. Methods

### 2.1. Study species and experimental animals

We used first-generation progeny of wild-caught European lobsters (*H. gammarus*), which were hatched under controlled conditions at the Ichthyogenic Experimental Marine Centre, University of Tuscia (Tarquinia, VT, Italy: 42.201851, 11.721749). Four ovigerous females were caught by local fishermen—August to November 2021—from two distinct locations along the Tyrrhenian coast (Porto Ercole, Italy: 42.393183, 11.211192; Montalto Marina, Italy: 42.327235, 11.572900) and transported to the research facilities. The females were housed individually in 1500 l tanks connected to a recirculating aquaculture system, which supplied seawater consistently during the five months until the eggs hatched. Females were fed daily with fish fillets alternating with thawed mussels. The seawater flowed through a suspended solid removal unit (63 µm mesh), a bio-filter reactor, a foam fractionator and a UV sterilizer before entering the holding tanks. Water temperature was set at 17°C, while salinity, dissolved oxygen, pH and nitrogen (NO_2_^−^ and NO_3_^−^) were monitored daily and kept within the optimal range for the species [36].

Daily, we retrieved from the bottom of each tank the eggs that had been lost by a female to assess their developmental stage. The newly hatched (planktonic) larvae were also collected daily, counted and housed separately for each female into a 200 l upwelling, aerated vessel at low stocking density, with a maximum age difference of 3 days between individuals, to minimize cannibalism (approx. 20 larvae l^−1^ [36–38]). The vessels were connected to the same recirculating system described above so that water conditions experienced by the newly hatched larvae reflected conditions during their incubation. Larvae were fed ad libitum with a mix of frozen *Artemia* sp., *Mysis* sp. and krill (*Euphasiidae* sp.).

### 2.2. Experimental procedure

**Figure 1.**
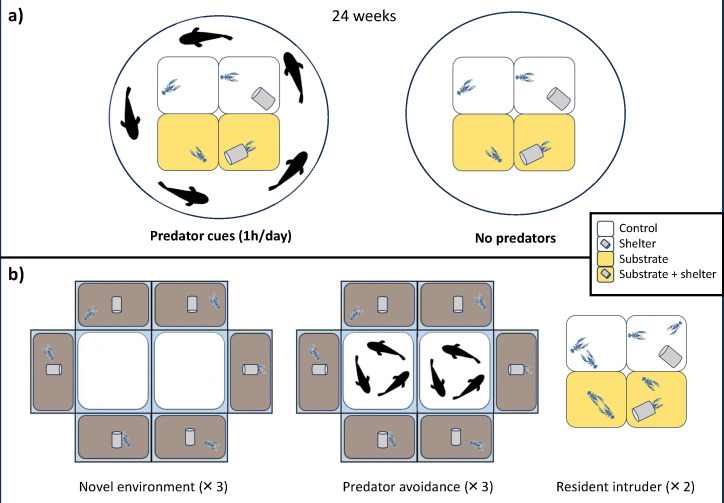
Representation of the experimental procedure. (a) Ontogenetic treatments (rearing and predator). Juveniles were exposed to different rearing treatments that differed in the presence/absence of environmental stimuli: shelter and substrate. Half of the animals were also exposed to predator cues (1 h per day) during development, while the others were not. Juveniles were exposed to these conditions from the beginning of their benthic life for approximately 20 consecutive weeks. (b) Behavioural tests: novel environment and predator avoidance. The set-up consisted of two squared, central areas to house the predators during the predator avoidance test and six rectangular compartments equipped with substrate and a shelter, in which the experimental animals were individually housed. The resident-intruder test was performed at the IX developmental stage on a subsample of the animals, in which an intruder was placed within an individual’s housing tank.

Approximately 14 days post hatching, the planktonic larvae metamorphosed into postlarvae (i.e. their IV developmental stage began [36]). Then, 256 postlarvae (64 per female) were randomly selected for the experiment and distributed across the experimental treatments, in which the animals were maintained for 20 consecutive weeks (i.e. 140 days), which corresponded to stages IV to IX of their development [36]. Specifically, four identical floating grids were set up for the experiment, each one placed inside a 1500 l tank. Each grid consisted of 64 individual square-shaped enclosures (8 cm per side and 3 cm deep each), which were made of solid plastic material with a perforated bottom surface (1 mm^2^) to ensure adequate water exchange between the individual enclosures and the housing tank. For each grid, individual tanks were randomly split across four rearing treatments that differed from each other for the presence/absence of a substrate (calcium carbonate gravel) and/or a shelter (PVC pipe): no substrate and no shelter (control), substrate, shelter, and substrate and shelter (figure 1a).

Individuals from two floating grids were also exposed to predator cues (predator treatment) for 1 h a day throughout the 20 experimental weeks (predator cues treatment; figure 1a). Each predator treatment tank hosted five juvenile sea breams (*Sparus aurata,* 10 ± 1 cm in length)—a common predator of small crustaceans [39]—for 1 h per day. Individuals in the predator treatment could perceive the presence of their predator via chemical cues [40], but the opaque, perforated material of the individual enclosures prevented visual interactions between individuals and their predators. After the 1 h exposure, predators were removed and the grids with the experimental lobsters were transferred into clean, predator-free holding tanks. On the contrary, animals housed in the two control grids were never exposed to predators (no predator treatment; figure 1a). Control grids were also moved into different, clean holding tanks once a day to balance the procedure across treatments.

Animals were fed daily with a mixture of frozen *Artemia* sp., *Mysis* sp. and frozen krill (*Euphasiidae* sp.) ad libitum. The light : dark cycle was set at 08.30 to 17.30 throughout the study, as per the natural conditions at the time of the study, and illumination was diffused through 36 W aquarium lamps.

### 2.3. Behavioural assays

The behavioural responses of the juveniles were scored, repeatedly over their ontogeny, across three different behavioural tests: novel environment, predator avoidance and resident-intruder test (figure 1b). Behavioural tests were always performed one week after the animals moulted to standardize the procedure, as suggested by Castro & Cobb [41]. To do so, the days between moults were monitored for each individual throughout the study.

The novel environment and predator avoidance tests were performed on each animal consecutively, in this order, in an experimental arena to minimize manipulation, for a total of three replicates per individual (approximately at 3, 6 and 10 weeks post hatching), corresponding to developmental stages IV, V and VI. Instead, the resident-intruder test was performed twice on each animal, within its individual’s rearing enclosure, at approximately 20 weeks post hatch, i.e. at the IX developmental stage.

Behavioural trials were recorded with a GoPro Full HD camera (48 frames s^−1^) and videos were scored, blind to the treatment, with the software Boris 5.1.3 [42].

#### 2.3.1. Novel environment and predator avoidance test

The experimental arena consisted of two central, squared tanks (21.5 cm per side and 11 cm high; predator areas) surrounded by six rectangular test tanks (21.5 cm long, 11 cm wide, and 11 cm high), each one equipped with gravel substrate and a shelter (figure 1b). Before the novel environment test started, we collected six experimental animals and placed them individually in the rectangular test tanks, inside their shelters (figure 1b). After 1 min, the doors of the shelters were simultaneously opened, and the lobsters were free to explore the novel environment for a total of 5 min.

Soon after the novel environment test ended, three juvenile seabreams (10 ± 1 cm in length) were released into each of the two squared, central tanks and the predator avoidance test was initiated (figure 1b). The behaviour of each individual lobster was recorded for a further 5 min while in the presence of the predators. Notably, transparent and perforated walls divided the predator arenas from the lobsters’ test tank, so that lobsters were able to both visually and chemically perceive the presence of their predators, which is known to increase the perceived predation risk in prey animals compared with when they are exposed to simpler, unimodal cues [43,44]. At the end of the predator avoidance test, juvenile lobsters were moved back into their housing containers and the test tanks were emptied and filled with clean water before the next trial commenced.

We chose activity (time spent moving, in seconds) and shelter use (time spent inside the shelter, in seconds) as the reference traits [45,46]. In fact, among-individual variation in these behaviours is a target of selection in non-sessile animals [26] and has both ecological and evolutionary consequences [47]. For instance, individuals who are more active and risk-prone than others might be more successful in securing resources, but at the cost of higher mortality.

#### 2.3.2. Resident-intruder test

The resident-intruder test was performed on 96 of the experimental individuals, randomly selected and balanced across treatments (figure 1b). Individuals were assayed twice, with tests 4 days apart, in their individual rearing containers. In particular, we measured aggressiveness (latency to attack an intruder, in seconds [48]). To do so, 50 juvenile lobsters were used as the intruders and were comparable in age and size to the focal individuals.

Before a resident-intruder trial started, the shelter was removed from the rearing containers belonging to the shelter and substrate + shelter treatments, and the selected intruder was placed inside a perforated transparent cylinder and located in the middle of the container. After 20 s the transparent cylinder was gently lifted, and the intruder was free to physically interact with the resident lobster for up to two consecutive minutes (figure 1b). We recorded the latency of the resident lobster to attack the intruder. To minimize distress in the animals during the trials, a test was terminated soon after the first attack was observed. Each intruder was changed after each trial, and a new intruder was randomly chosen so that focal animals never encountered the same intruder twice.

### 2.4. Morphometric measurements

Each juvenile was photographed with a Fujifilm XP140 camera and its carapace length (in mm) was measured with the dedicated software ImageJ [49], blind to the treatment, before each round of behavioural tests. Therefore, we collected three morphometric datapoints per individual. Additionally, we determined the time between two consecutive moults (i.e. intermoult period, in days) for each individual.

### 2.5. Data analysis

A final sample size of 250 juvenile lobsters (control: *n* = 63; substrate: *n* = 61; shelter: *n* = 62; substrate + shelter: *n* = 64) out of 256 individuals completed all behavioural and morphometric assays—six juveniles were lost due to early mortality—for a total of 1536 behavioural observations. Instead, 96 individuals performed the resident-intruder test, for a total of 192 behavioural observations. Overall, our sample size relied on greater than 130 h of video recordings.

Data analysis and figures preparation were performed with RStudio v. 4.2.1 [50], using the packages *lmerTest, emmeans* and *ggplot2* [51–53]. We assumed Gaussian error distribution, which was confirmed for all response variables after visual inspection of model residuals. The significance level was set at α < 0.05.

We were interested in testing whether and how the presence of environmental stimuli during development altered mean behaviours and life-history traits of the animals, as well as their individual- and group-level plasticity in behaviour. To do so, we fitted generalized linear mixed-effects models (LMMs) with activity (i.e. time spent moving, in seconds), shelter use (i.e. time spent inside the shelter, in seconds), body size (i.e. carapace length, in mm) and intermoult period (i.e. time between two consecutive moults, in days) as the dependent variables, respectively. In the fixed-effect structure of the models for activity and shelter use, we included rearing treatment (presence/absence of a shelter and/or substrate; four levels), predator treatment (predator cues and no predators; two levels), test (novel environment and predator avoidance; two levels), grid (four levels), mother ID (four levels), stage (three levels), test duration (in seconds) and the interaction between rearing treatment, predator treatment and test. In the aggressiveness model, we included rearing and predator treatment, their interaction, grid and mother ID as the fixed effects. The models for carapace length and intermoult period included, instead, rearing treatment, predator treatment, stage, their (three-way) interaction, grid and mother ID as the fixed effects. We ran pairwise comparisons with the conservative Bonferroni method for significant predictors while accounting for the variation explained by other predictors.

We included individual identities (random intercepts) in the random structure of all models to account for repeated measures. The aggressiveness model also included intruder identities as random intercepts. Since individuals might differ in the way that they adjusted their activity and shelter use across tests, developmental stages and treatments, or as function of their mother ID, we also tested for the presence of heterogeneous variance between tests, developmental stages, rearing treatments, predator treatments and mother IDs (i.e. random slopes/regressions). We did this by running five separate models for activity and shelter use in which we included random intercepts and slopes one by one. Then, we used both likelihood ratio tests and Akaike information criteria (AIC) to compare models with different random slopes and chose models with the best likelihood ratio and lower AIC. In addition, we inspected whether random intercepts explained a relatively large portion of the variation observed, i.e. whether individual variation in activity and shelter use was highly structured [54].

## 3. Results

**Figure 2.**
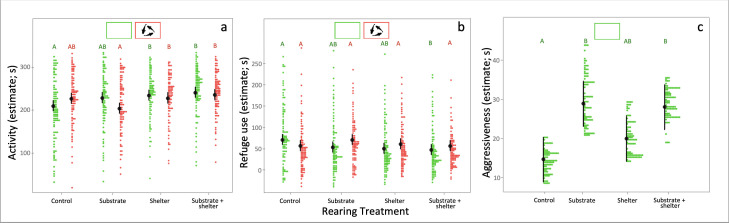
Effects of the rearing and predator treatments experienced during developments on the behavioural traits. In (a) and (b) are, respectively, presented the effects of both rearing and predator treatments on activity and shelter (refuge) use, and in (c) the effect of rearing treatment on aggressiveness. Estimated marginal means and their confidence intervals are represented in black, while datapoints are represented with coloured dots. In each predator treatment, plots that do not share the same letter are significantly different.

The rearing conditions to which individuals were exposed during their early development had a strong effect on the activity levels and the aggressiveness of the animals, with marginal, albeit non-significant, effects also on the growth rate of the individuals (i.e. intermoult period; table 1). In particular, the presence of a shelter in the housing enclosure resulted in individuals being generally more active than their siblings raised under both control conditions and in the presence of substrate only (figure 2a). Instead, juveniles raised in enclosures with gravel substrate were less aggressive towards intruders (i.e. longer latencies to attack) than conspecifics raised under control conditions (figure 2c). On the contrary, no consistent effects of either substrate or shelter availability were observed for shelter use (figure 2b).

**Table 1.** Results from the fixed-factor structure of the generalized LMMs. Rearing treatment (four levels), predator treatment (two levels), test (two levels), grid (four levels), mother ID (four levels), stage (three levels), test duration (in seconds) and the interaction between rearing treatment, predator treatment and test are included as fixed effects in the models for activity and shelter use. In the aggressiveness model, rearing treatment, predator treatment, their interaction, grid and mother ID were the fixed effects. For the life-history traits carapace length and intermoult period, rearing treatment, predator treatment, stage, their (three way) interaction, grid and mother ID are included as the fixed effects. Significance was α < 0.05, and significant results are in bold.

		mean sq.	d.f.	*F*	*p*-value
activity	rearing treatment	24 551	3, 397	5.464	**<0.001**
	predator treatment	1302	1, 428	0.290	0.590
	test	1 645 111	1, 483	366.714	**<0.001**
	grid	8847	2, 453	1.972	0.140
	mother ID	2793	3, 454	0.623	0.600
	stage	346 274	2, 537	77.188	**<0.001**
	duration	26 743	1, 1281	5.961	**0.015**
	rearing × predator treatment	15 704	3, 398	3.500	**0.016**
	rearing treatment × test	24 764	3, 805	5.520	**<0.001**
	predator treatment × test	581	1, 804	0.130	0.719
	rearing × predator × test	6346	3, 806	1.414	0.237
shelter use	rearing treatment	6955	3, 481	1.666	0.173
	predator treatment	568	1, 510	0.136	0.712
	test	660 251	1, 472	158.204	**<0.001**
	grid	2127	2, 531	0.510	0.601
	mother ID	2317	3, 532	0.555	0.645
	stage	24 366	2, 518	72.406	**<0.001**
	duration	26 743	1, 1280	5.838	**0.016**
	rearing × predator treatment	10 540	3, 481	2.526	0.056
	rearing treatment × test	10 066	3, 890	2.412	0.065
	predator treatment × test	132	1, 889	0.032	0.860
	rearing × predator × test	3406	3, 891	0.816	0.485
aggressiveness	rearing treatment	2267.42	3, 84	5.485	**<0.001**
	predator treatment	4.64	1, 85	0.011	0.916
	grid	637.45	2, 56	1.542	0.222
	mother ID	378.46	3, 80	0.915	0.437
	rearing × predator	338.06	3, 81	0.818	0.488
carapace length	rearing treatment	0.360	3, 244	1.128	0.334
	predator treatment	4.100	1, 244	12.901	**<0.001**
	grid	0.590	2, 244	1.854	0.156
	mother ID	10.220	3, 244	32.187	**<0.001**
	stage	839.420	2, 491	2644.197	**<0.001**
	rearing × predator treatment	0.330	3, 244	1.039	0.376
	rearing treatment × stage	0.550	6, 491	1.744	0.110
	predator treatment × stage	0.170	2, 491	0.537	0.585
	rearing × predator × stage	0.150	6, 491	0.485	0.819
intermoult period	rearing treatment	13.340	3, 237	2.436	0.065
	predator treatment	0.090	1, 237	0.017	0.896
	grid	7.650	2, 237	1.396	0.249
	mother ID	4.350	3, 237	0.793	0.499
	stage	1933.660	1, 242	353.033	**<0.001**
	rearing × predator treatment	13.130	3, 237	2.397	0.069
	rearing treatment × stage	37.130	3, 242	6.779	**<0.001**
	predator treatment × stage	0.99	1, 242	0.181	0.670
	rearing × predator × stage	10.27	3, 242	1.875	0.134

**Figure 3.**
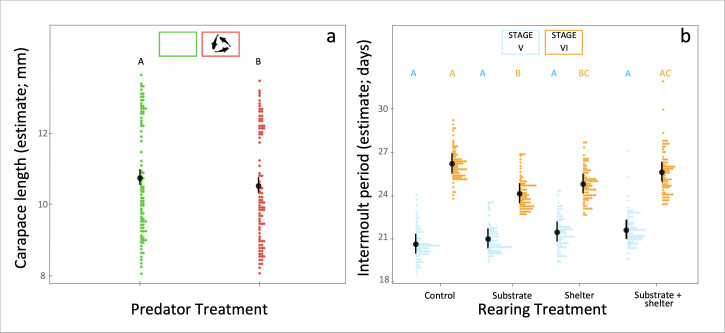
Effects of the rearing and predator treatments experienced during developments on the life-history traits. In (a) and (b) are, respectively, presented the effect of predator treatment on carapace length and the effect of rearing treatment on the intermoult period. Estimated marginal means and their confidence intervals are represented in black, while datapoints are represented with coloured dots. In each predator treatment (a) or stage (b), plots that do not share the same letter are significantly different.

The exposure to the predator cues during development had a negative impact on the carapace length of the individuals, with juveniles from the predator treatment being on average smaller than those not exposed to predator cues (figure 3a). Overall effects of predator exposure were less evident on the other traits. However, we observed that the interaction between rearing conditions and predator cues explained a large portion of the behavioural variance observed (table 1). For instance, juveniles that had access to shelters during development were generally more active and hid into their shelter less than control individuals, but this was true only for animals not exposed to predator cues during development (figure 2a,b). On the contrary, when individuals were exposed to predator cues during development such an effect varied (figure 2a) or even disappeared (figure 2b).

The behavioural and life-history traits of the individuals varied, on average, across ontogenetic stages (table 1; figure 3b). For instance, activity decreased and shelter use increased over development (table 1). We also observed that intermoult period (i.e. growth rate) varied between ontogenetic stages depending on the rearing treatment experienced by the individuals (figure 3b): the time between two consecutive moults did not differ between rearing treatments early in life (i.e. stage IV to V), but individuals reared in the presence of either substrate or a shelter—but not both substrate and shelter together—grew faster than control animals later in life (i.e. stage V to VI; figure 3b).

As expected, activity levels and shelter use varied between tests (novel environment and predator avoidance; table 1), so that individuals spent on average less time exploring the experimental arena and hid longer when live predators were present than in the absence of perceived predation risk. However, the ability of the animals to plastically adjust their activity levels across contexts differed substantially between rearing treatments, and a similar, although marginally non-significant, effect was also found for shelter use (table 1). In particular, activity levels did not vary between rearing treatments in the absence of predators, i.e. novel environment test (control–substrate: estimate ± s.e. *=* −0.97 ± 7.32, *p* = 0.09; control–shelter: estimate ± s.e. *=* 0.78 ± 7.31, *p* = 0.09; control–substrate + shelter: estimate ± s.e. *=* −7.24 ± 7.27, *p* = 0.09; substrate–shelter: estimate ± s.e. *=* 1.76 ± 7.34, *p* = 0.09; substrate–substrate + shelter: estimate ± s.e. *=* −6.26 ± 7.31, *p* = 0.09; shelter–substrate + shelter: estimate ± s.e. *=* −8.02 ± 7.29, *p* = 0.09). In sharp contrast, individuals that had access to shelters during development were more active under perceived predation risk than their counterparts raised with substrate only or deprived of both shelter and substrate, i.e. predator avoidance test (control–substrate: estimate ± s.e. *=* 5.40 ± 8.98, *p* = 0.09; control–shelter: estimate ± s.e. *=* −26.35 ± 8.97, *p* = 0.02; control–substrate + shelter: estimate ± s.e. *=* −32.40 ± 8.94, *p* < 0.01; substrate–shelter: estimate ± s.e. *=* −31.74 ± 9.00, *p* < 0.01; substrate–substrate + shelter: estimate ± s.e. *=* −37.80 ± 8.97, *p* < 0.01; shelter–substrate + shelter: estimate ± s.e.*=* −6.05 ± 8.95, *p* = 0.09). Differently, the ability of the animals to plastically adjust their behaviours across tests did not vary depending on whether they were exposed to perceived predation risk during development (table 1), although variation was only marginally non-significant (*p* = 0.053 and *p* = 0.051, respectively).

We found evidence that the inclusion of test and stage as random slopes increased the model fit for activity and shelter use (table 2). In other words, individuals differed in behaviour among each other in the way that they adjusted their activity levels and shelter use across tests and developmental stages (i.e. behavioural plasticity). Instead, no differences were detected between models with rearing treatment or mother IDs as the random slopes, albeit the random slope predator treatment was marginally non-significant (table 2). Therefore, we did not retain these terms in our final models. We also detected that the random intercepts (i.e. individual IDs) characterized a large portion of the behavioural variance observed (table 3), indicating that behavioural differences among individuals were relatively consistent over time (i.e. behavioural individuality was highly structured).

**Table 2.** Comparison of LMMs with different random (co)variance structures for activity (time spent moving) and shelter use (time spent inside the shelter). We compared two models with both random intercepts (ID) and slopes (test, rearing treatment, predator treatment, mother ID or stage) against a reduced model with only random intercepts. We also compared the model with random intercepts with a model in which random intercepts were excluded. Significant improvements in the model fit are tested with both AIC and likelihood ratio tests (*p*). Rearing treatment (control, substrate, shelter, substrate + shelter), predator treatment (predator cues, no predators), grid (four levels), mother ID (four levels), stage (three levels), test duration (in seconds) are included as fixed effects in models considered for activity and shelter use. Significance was set at α < 0.05, and significant results are in bold; the model with random intercepts and test and stage as the random slopes was best supported as the most parsimonious model for activity, while random intercepts and stage as the random slopes explained a significant portion of the variance in shelter use. The random structure is written in the syntax of the *lmer R* package.

		χ^2^	AIC	*p*-value
activity	(test|ID)	29.506	25	**<0.001**
	(predator treatment|ID)	5.891	2	0.053
	(rearing treatment |ID)	2.222	16	0.987
	(mother ID|ID)	3.742	14	0.927
	(stage|ID)	108.000	104	**<0.001**
shelter use	(test|ID)	29.506	25	**<0.001**
	(predator treatment|ID)	5.979	2	0.051
	(rearing treatment|ID)	6.488	12	0.690
	(mother ID|ID)	13.169	5	0.155
	(stage|ID)	175.110	171	**<0.001**

**Table 3.** Results from the random-factor structure of the generalized LMMs. Models refer to the behaviours activity, shelter use, and aggressiveness and the life-history traits carapace length and intermoult period.

	variance between ± s.e.	test ± s.e.	stage ± s.e.	residuals ± s.e.
activity	1004.6 ± 1.9	665.6 ± 1.6	4003.4 ± 3.9	4486.1 ± 4.2
shelter use	1180.1 ± 2.1	714.3 ± 1.7	5394.9 ± 4.6	4173.4 ± 4.0
aggressiveness	22.1 ± 0.5	—	—	413.4 ± 2.0
carapace length	0.2 ± <0.1	—	—	0.3 ± <0.1
intermoult period	2.2 ± 0.1	—	—	5.5 ± 0.1

## 4. Discussion

Our analysis provides solid evidence that early-life exposure to complex rearing environments and cues from natural predators have important effects on the development of both average and individual-level behaviours and life-history traits in juvenile European lobsters. For instance, the presence of a shelter in the enclosure resulted in higher activity rates only in the individuals exposed to predator cues during development, but not in predator-free animals. The presence of a gravel substrate, instead, reduced aggressiveness towards intruders. Individuals reared with either substrate or a shelter grew faster than controls, while the exposure to predator cues during development altered the behavioural plasticity of the animals and also resulted in lobsters that were smaller in size.

A main result of this work is that gravel substrate and a shelter in the rearing enclosures resulted in higher activity levels and lower aggressiveness in the animals. Studies on other aquatic organisms indicate that the presence of such environmental enrichments promotes muscle development and enhances the neural plasticity of the animals by increasing their dopaminergic and serotonergic activity that, in turn, promotes exploratory behaviours, spatial orientation and learning capacities [55–57]. Gravel substrate has also been shown to elicit the selective use of claws in captive-reared lobsters, thereby promoting claws asymmetry, which plays a key role for feeding and mate choice in this species [34,58–60]. In line with our results, Torrezani and collaborators [61] also observed reduced aggressiveness in captive-bred fish (*Tilapia rendaIli*) raised in the presence of gravel substrate and shelter. In fact, it is reasonable to believe that habitat complexity provides the opportunity for individuals to establish their territories and safe refugia, thereby reducing stress levels and attenuating aggressive behaviours [62]. This is important because the availability of substrate and shelters in the housing enclosure has been shown to also increase the survival of European lobsters when released into semi-natural conditions [31,32]. In line with this interpretation, our lobsters raised in the presence of substrate and/or shelter grew faster (i.e. lower intermoult periods) than control groups. Although, to the best of our knowledge, there are no studies that have explored the effects of environmental enrichments on the intermoult period of lobsters, existing research on aquatic organisms indicates that enriched environments reduce stress levels and favour lower resting metabolic rates, which, in turn, are likely to reduce the energy expenditure for body maintenance and promote growth [20,62–65]. It is therefore reasonable to assume that environmental enrichment has contributed, over the course of development, to a decrease in the stress levels and, consequently, to higher growth rates in our animals. Notably, we observed a consistent pattern of longer shelter use and lower activity levels with advancing age, suggesting a shift towards more pronounced benthic attitudes and lower risk taking with advancing age, which is well documented for these animals [33,41,66,67].

Our work points out that the exposure to predatory cues during development results in smaller body sizes of the individuals. According to classic life-history theory, in which predatory regimes typically drive the life-history strategies of animal populations [68], a slower growth can be interpreted as a trade-off between securing resources and reducing risks of being predated [69–71]. In fact, prior evidence indicates that prey tend to reduce activity levels and increase shelter use in response to the presence of a predator, therefore reducing also the time and effort available for feeding [72–74]. In support of this interpretation, we observed that individuals spent on average more time hiding in the shelter in the presence of perceived predation risk (predator avoidance test) than when predators were absent (novel environment test).

Evidence from a recent review indicates that the effects from different environmental enrichments can interact with one another so that concurrent effects from complex environmental conditions might explain the discrepancies that are frequently observed in the literature (reviewed in [4]). For instance, the interaction between the exposure to environmental enrichments, i.e. the presence of plants and rocks, and live prey enhanced foraging success on novel prey in hatchery-reared Atlantic salmons, exceeding the benefits of either strategy alone [75]. Additionally, environmental enrichment was shown to interact with the density of Atlantic salmons, negatively impacting both growth and post-release survival at higher densities [76]. Our work adds onto this literature, showing that substrate and shelter availability promote activity and risk taking in lobsters, but that such effects taper away when animals are simultaneously exposed to predatory cues during development.

Our analysis reveals that effects from shelter availability and perceived predation risk during development interact with each other also in determining the behavioural plasticity of lobsters later in their life. In fact, individuals that had access to shelters during their development were on average more active than others during the predator avoidance test, but not when they were tested in the novel, predator-free test. Such group-level and treatment-dependent variation in the behavioural plasticity of the lobster is likely to impact their ability to survive in the wild, with plasticity boosting the long-term persistence of animal groups in general [15]. In fact, theory predicts that shelter-seeking animals should strategically adjust their antipredator responses to survive predation risk while also minimizing the substantial costs associated with lost foraging opportunities [72,74,77]. Therefore, rearing conditions that promote the development of group-level plasticity in key behaviours should be preferred if we are to improve the effectiveness of lobsters’ conservation programmes.

Ultimately, our results revealed that lobsters displayed substantial differences in the way that individuals adjusted their behaviour across developmental stages and behavioural tests (i.e. individual-level behavioural plasticity). Individual-level plasticity in activity and shelter use also tended to differ between individuals exposed to predator cues during development and control animals, although the differences were marginally non-significant. These findings indicate that the early-life environment can play an important role in shaping phenotypic variation also within animal groups and potentially impact their ability to respond to rapid environmental changes [14,78]. We know that behavioural variation among individuals is key for populations to persist in the face of global changes (e.g. [15,46]). Yet it is costly for an organism to maintain the sensory machinery needed for high responsiveness (reviewed in [79]) and reducing behavioural plasticity should be adaptive if predation pressures are low [80]. In support of this interpretation, classic studies indicate that behavioural plasticity in prey species increases with increasing predation risk (e.g. [81,82]), as unpredictable behaviours help individuals to lower their susceptibility to predation [83]. So it is reasonable to assume that environmental stimuli experienced early in life can be important to fuel plasticity in ecologically relevant behaviours of the lobsters and enhance the likelihood of their post-release success. While it remains unknown whether these experimentally induced phenotypic variations will persist over time and after animals are released into the wild, the literature suggests that individual-level measurements performed in the laboratory well reflect the natural variation observed among the individuals once released into the wild [84].

A key goal in conservation aquaculture is producing animals that can effectively survive and reproduce in the wild. We show that introducing ecologically relevant stimuli in the rearing environment promotes population- and individual-level adaptations in key behaviours and life-history traits of the animals, which are essential for thriving under challenging environmental conditions. Therefore, we propose that the improvement of aquaculture technologies is critical for expanding the scope of aquaculture practices to conservation purposes, with the goal of contrasting the decline of natural resources and providing solutions for their management.

## Data Availability

Data and the R code used for the statistical analysis and figures preparation of this work are available at https://figshare.com/s/0a75a10777d0ab9415db.
